# Supplementary material for: Analysis of bacterial vaginosis, the vaginal microbiome, and sexually transmitted infections following the provision of menstrual cups in Kenyan schools: Results of a nested study within a cluster randomized controlled trial
Source: PLoS Med. 2023 Jul 25;20(7):e1004258. doi: 10.1371/journal.pmed.1004258 (PMC10368270; doi:10.1371/journal.pmed.1004258)
Supplement: S1 CONSORT Checklist — (PDF) [file pmed.1004258.s001.pdf]

# CONSORT 2010 checklist of information to include when reporting a randomised trial

| Section/Topic | Item No | Checklist item | Reported on page No |
|---|---|---|---|
| **Title and abstract** | | | |
| | 1a | Identification as a randomised trial in the title | Title page |
| | 1b | Structured summary of trial design, methods, results, and conclusions (for specific guidance see CONSORT for abstracts) | Abstract |
| **Introduction** | | | |
| Background and objectives | 2a | Scientific background and explanation of rationale | Intro, Paras 1-2 |
| | 2b | Specific objectives or hypotheses | Intro, Para 3 |
| **Methods** | | | |
| Trial design | 3a | Description of trial design (such as parallel, factorial) including allocation ratio | Methods, Para 2 |
| | 3b | Important changes to methods after trial commencement (such as eligibility criteria), with reasons | Methods, Para 13 |
| Participants | 4a | Eligibility criteria for participants | Methods, Para 13 |
| | 4b | Settings and locations where the data were collected | Methods, Para 2 |
| Interventions | 5 | The interventions for each group with sufficient details to allow replication, including how and when they were actually administered | Methods, Para 2 |
| Outcomes | 6a | Completely defined pre-specified primary and secondary outcome measures, including how and when they were assessed | Methods, Para 9-10 |
| | 6b | Any changes to trial outcomes after the trial commenced, with reasons | NA |
| Sample size | 7a | How sample size was determined | Methods, Para 4 |
| | 7b | When applicable, explanation of any interim analyses and stopping guidelines | NA |
| Randomisation: | | | |
| Sequence generation | 8a | Method used to generate the random allocation sequence | Methods, Para 2 |
| | 8b | Type of randomisation; details of any restriction (such as blocking and block size) | Methods, Para 2 |
| Allocation concealment mechanism | 9 | Mechanism used to implement the random allocation sequence (such as sequentially numbered containers), describing any steps taken to conceal the sequence until interventions were assigned | Methods, Para 2 |
| Implementation | 10 | Who generated the random allocation sequence, who enrolled participants, and who assigned participants to interventions | Methods, Para 2 |
| Blinding | 11a | If done, who was blinded after assignment to interventions (for example, participants, care providers, those | Mehtods, Para 2, 9 |

| | | | |
|---|---|---|---|
| | | assessing outcomes) and how | Methods, Para 2 |
| | 11b | If relevant, description of the similarity of interventions | NA |
| Statistical methods | 12a | Statistical methods used to compare groups for primary and secondary outcomes | Methods, Paras 9-11 |
| | 12b | Methods for additional analyses, such as subgroup analyses and adjusted analyses | Methods, Paras 12-13 |
| **Results** | | | |
| Participant flow (a diagram is strongly recommended) | 13a | For each group, the numbers of participants who were randomly assigned, received intended treatment, and were analysed for the primary outcome | Results, Para 1, consort diagram |
| | 13b | For each group, losses and exclusions after randomisation, together with reasons | Consort diagram |
| Recruitment | 14a | Dates defining the periods of recruitment and follow-up | Results, Para 1 |
| | 14b | Why the trial ended or was stopped | NA - completed |
| Baseline data | 15 | A table showing baseline demographic and clinical characteristics for each group | Table 1 |
| Numbers analysed | 16 | For each group, number of participants (denominator) included in each analysis and whether the analysis was by original assigned groups | Tables 2-4 |
| Outcomes and estimation | 17a | For each primary and secondary outcome, results for each group, and the estimated effect size and its precision (such as 95% confidence interval) | Results, Para 3, Table 3 |
| | 17b | For binary outcomes, presentation of both absolute and relative effect sizes is recommended | Results, Para 3, Table 3 |
| Ancillary analyses | 18 | Results of any other analyses performed, including subgroup analyses and adjusted analyses, distinguishing pre-specified from exploratory | Results, Para 4-5 |
| Harms | 19 | All important harms or unintended effects in each group (for specific guidance see CONSORT for harms) | Results, Para 6 |
| **Discussion** | | | |
| Limitations | 20 | Trial limitations, addressing sources of potential bias, imprecision, and, if relevant, multiplicity of analyses | Discussion, Para 5 |
| Generalisability | 21 | Generalisability (external validity, applicability) of the trial findings | Discussion, Para 5 |
| Interpretation | 22 | Interpretation consistent with results, balancing benefits and harms, and considering other relevant evidence | Discussion, Paras 1-5 |
| **Other information** | | | |
| Registration | 23 | Registration number and name of trial registry | Abstract; Methods, Para 2 |
| Protocol | 24 | Where the full trial protocol can be accessed, if available | Abstract, Methods, Para 2 |
| Funding | 25 | Sources of funding and other support (such as supply of drugs), role of funders | Title Page, Funding Source & Role |

# *CONSERVE Checklist*

| **CONSERVE-CONSORT Extension: [DATE]** | | | |
|---|---|---|---|
| **Item** | **Item Title** | **Description** | **Page No.** |
| I. | Extenuating Circumstances | Describe the circumstances and how they constitute extenuating circumstances. | Methods Para 2 |
| II. | Important Modifications | a. Describe how the modifications are important modifications. | Methods Para 12 |
| | | b. Describe the impacts and mitigating strategies, including their rationale and implications for the trial. | (see below) Methods, para 12 |
| | | c. Provide a modification timeline. | Methods, Para 2 |
| III. | Responsible Parties | State who planned, reviewed and approved the modifications. | Methods, Para 2 |
| IV. | Interim data | If modifications were informed by trial data, describe how the interim data were used, including whether they were examined by study group, and whether the individuals reviewing the data were blinded to the treatment allocation. | NA |

| **CONSORT Number and Item** | | For each row, if important modifications occurred check "direct impact" and/or "mitigating strategy" and describe the changes in the trial manuscript or supplement. Check "no change" for items that are unaffected in the extenuating circumstance. | | | **Page No.** |
|---|---|---|---|---|---|
| | | **No Change** | **Impact*** | **Mitigating Strategy**** | |
| 1 | Title and abstract | X | | | |
| 2 | Introduction | X | | | |
| 3 | Methods: Trial Design | | X | Sensitivity analysis excluding data from study visit occurring during COVID-19 | Methods, Para 12 |
| 4 | Methods: Participants | X | | | |
| 5 | Methods: Interventions | X | | | |
| 6 | Methods: Outcomes | X | | | |
| 7 | Methods: Sample Size | X | | | |
| 8-10 | Methods: Randomisation | X | | | |

| | | | | | |
|---|---|---|---|---|---|
| 11 | Methods: Blinding | X | | | |
| 12 | Methods: Statistical methods | | X | Sensitivity analysis excluding data from study visit occurring during COVID-19 | Methods, Para 12 |
| 13 | Results: Participant flow | X | | | |
| 14 | Results: Recruitment | X | | | |
| 15 | Results: Baseline data | X | | | |
| 16 | Results: Numbers analysed | | X | Sensitivity analysis excluding data from study visit occurring during COVID-19 | Methods, Para 12 |
| 17 | Results: Outcomes and estimation | | X | Sensitivity analysis excluding data from study visit occurring during COVID-19 | Methods, Para 12 |
| 18 | Results: Ancillary analyses | X | | | |
| 19 | Results: Harms | X | | | |
| 20 | Discussion: Limitations | X | | | |
| 21 | Discussion: Generalisability | X | | | |
| 22 | Other information: Registration | X | | | |
| 23 | Other information: Protocol | | X | Sensitivity analysis excluding data from study visit occurring during COVID-19 | Methods, Para 12 |
| 24 | Other information: Funding | X | | | |

*Aspects of the trial that are directly affected or changed by the extenuating circumstance and are not under the control of investigators, sponsor or funder.
**Aspects of the trial that are modified by the study investigators, sponsor or funder to respond to the extenuating circumstance or manage the direct impacts on the trial.

The CONSERVE-CONSORT Checklist is licensed by the CONSERVE Group under the Creative Commons Attribution-NonCommercial-NoDerivs 4.0 International license.
