# Supplementary figures and images for: Analysis of bacterial vaginosis, the vaginal microbiome, and sexually transmitted infections following the provision of menstrual cups in Kenyan schools: Results of a nested study within a cluster randomized controlled trial
Source: PLoS Med. 2023 Jul 25;20(7):e1004258. doi: 10.1371/journal.pmed.1004258 (PMC10368270; doi:10.1371/journal.pmed.1004258)

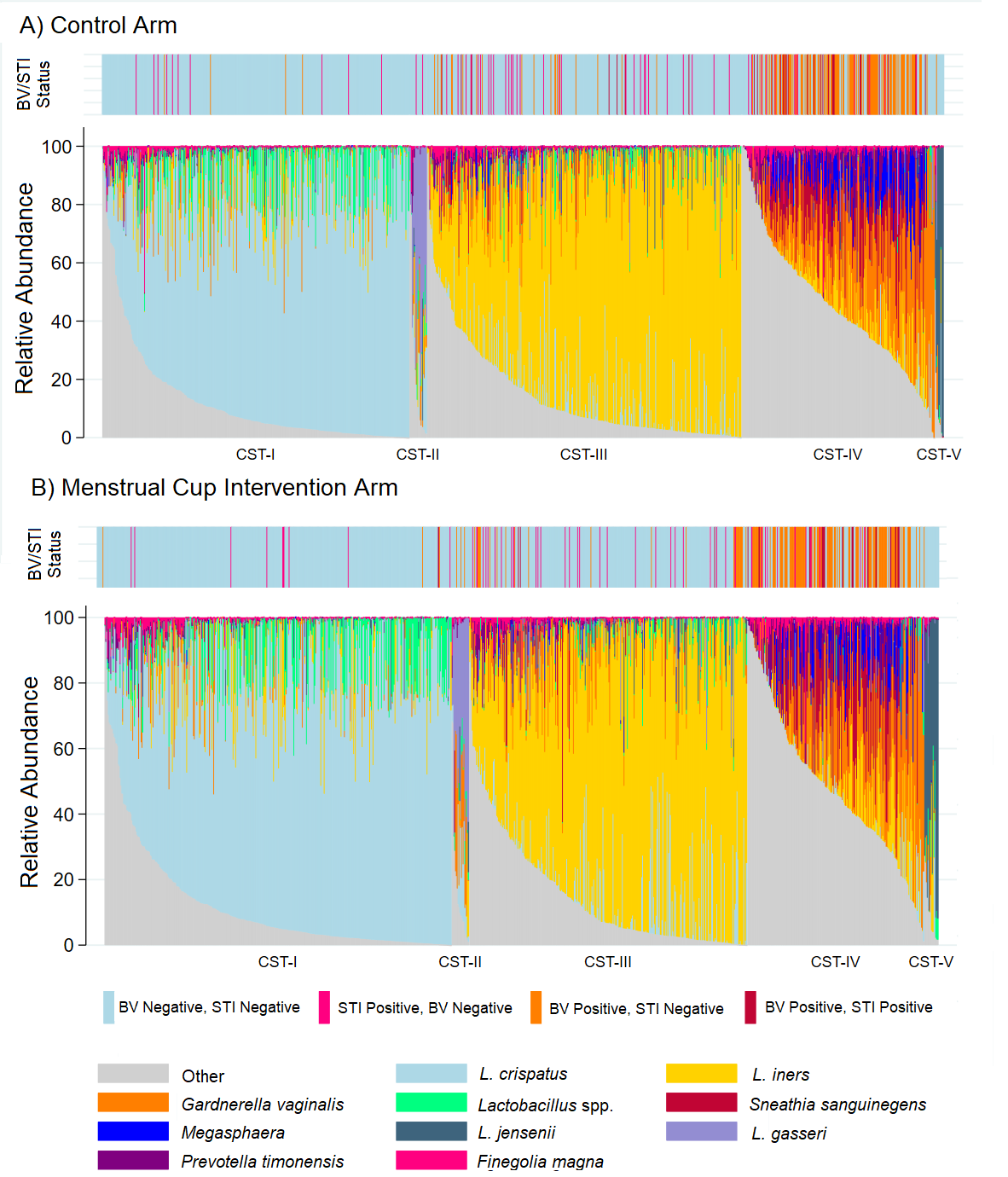

Supplement: S1 Fig — The relative abundance of the 10 taxa with the highest mean relative abundance is shown (y-axis), separated by CST (x-axis) with individual subjects represented by individual bars for observations from (A) control arm participants and (B) intervention arm participants. The bar at the top of each graph represents the presence of BV and/or STI for each observation. BV, bacterial vaginosis; CST, community state type; STI, sexually transmitted infection. (TIF) [file pmed.1004258.s002.tif]
