# Supplementary material for: Analysis of bacterial vaginosis, the vaginal microbiome, and sexually transmitted infections following the provision of menstrual cups in Kenyan schools: Results of a nested study within a cluster randomized controlled trial
Source: PLoS Med. 2023 Jul 25;20(7):e1004258. doi: 10.1371/journal.pmed.1004258 (PMC10368270; doi:10.1371/journal.pmed.1004258)
Supplement: S1 Table — (DOCX) [file pmed.1004258.s003.docx]

**S1 Table. Number of tests conducted, infections detected, and documented antimicrobial treatment by study time point.**

| Study Visit | Baseline | | | 6 Months | | | 12 Months | | | 18 Months | | | 30 Months | | |
| --- | --- | --- | --- | --- | --- | --- | --- | --- | --- | --- | --- | --- | --- | --- | --- |
|  | No. tested | No. detected | No.  treated | No. tested | No. detected | No.  treated | No. tested | No. detected | No.  treated | No. tested | No. detected | No.  treated | No. tested | No. detected | No.  treated |
| Bacterial vaginosis* | 436 | 49 | 48 | 424 | 39 | 37 | 395 | 57 | 55 | 398 | 56 | 56 | 395 | 88 | 81 |
| *Chlamydia trachomatis* | 436 | 27 | 25 |  |  |  | 395 | 26 | 26 |  |  |  | 395 | 45 | 44 |
| *Neisseria gonorrhoeae* | 436 | 6 | 6 |  |  |  | 395 | 12 | 12 |  |  |  | 395 | 8 | 6 |
| *Trichomonas vaginalis* | 436 | 13 | 13 |  |  |  | 395 | 13 | 12 |  |  |  | 395 | 20 | 15 |

“No.” = Number

*Due to unavailability of tinidazole, metronidazole (regimen) was given to treat BV for: 1 participant at 12 month visit, 1 participant at 18 month visit, 4 participants at 30 month visit
