# Supplementary material for: Analysis of bacterial vaginosis, the vaginal microbiome, and sexually transmitted infections following the provision of menstrual cups in Kenyan schools: Results of a nested study within a cluster randomized controlled trial
Source: PLoS Med. 2023 Jul 25;20(7):e1004258. doi: 10.1371/journal.pmed.1004258 (PMC10368270; doi:10.1371/journal.pmed.1004258)
Supplement: S2 Table — (DOCX) [file pmed.1004258.s004.docx]

**S2 Table. Cumulative Number of BV and STI cases by Study Arm.**

|  | **Control Arm*** | **Menstrual Cups Arm*** | **Total** |
| --- | --- | --- | --- |
|  | **N=221** | **N=211** | **N=432** |
|  | **n (%)** | **n (%)** | **n (%)** |
| **Bacterial vaginosis** |  |  |  |
| 0 occurrence | 133 (60.2) | 132 (62.6) | 265 (61.3) |
| 1 occurrence | 47 (21.3) | 51 (24.2) | 98 (22.7) |
| 2 occurrences | 24 (10.9) | 15 (7.1) | 39 (9.0) |
| 3 occurrences | 7 (3.2) | 7 (3.3) | 14 (3.2) |
| 4 occurrences | 6 (2.7) | 6 (2.8) | 12 (2.8) |
| 5 occurrences | 4 (1.8) | 0 (0.0) | 4 (0.9) |
| **Sexually transmitted infection** |  |  |  |
| 0 occurrence | 157 (71.0) | 159 (75.4) | 316 (73.2) |
| 1 occurrence | 47 (21.3) | 40 (19.0) | 87 (20.1) |
| 2 occurrences | 14 (6.3) | 9 (4.3) | 23 (5.3) |
| 3 occurrences | 3 (1.4) | 3 (1.4) | 6 (1.4) |

*Excludes the n=4 participants (n=2 in control arm; n=2 in menstrual cup arm) who did not provide specimens for BV, VMB, and STI testing subsequent to baselin
