# Supplementary material for: Analysis of bacterial vaginosis, the vaginal microbiome, and sexually transmitted infections following the provision of menstrual cups in Kenyan schools: Results of a nested study within a cluster randomized controlled trial
Source: PLoS Med. 2023 Jul 25;20(7):e1004258. doi: 10.1371/journal.pmed.1004258 (PMC10368270; doi:10.1371/journal.pmed.1004258)
Supplement: S1 Statistical Analysis Plan — (PDF) [file pmed.1004258.s005.pdf]

Menstrual cups, maturation of the adolescent vaginal microbiome, and STI/HIV risk
Funded by NIH, NICHD HD093780

STATISTICAL ANALYSIS PLAN

Version 1.0/NOV2021

CCG **Trial registration number:** ClinicalTrials.gov: NCT03051789

Protocol version and date: Version10.1- 31 July 2021

Chief Investigator: Dr. Supriya Mehta
Trial statisticians: Dr. Runa Bhaumik
SAP authors: Dr Runa Bhaumik, Dr. Supriya Mehta

SAP version history

Version Date	SAP Version #	Details of Changes
10-Nov-2021	1.0	NA

**Signatures**		
	Signature	Date
Runa Bhaumik	Runa Bhaumik	14Dec21
Supriya Mehta	Supriya Mehta	14Dec21
Duolao Wang	Duolao Wang	15Dec21

TABLE OF CONTENTS

ABBREVIATIONS 5
1. INTRODUCTION 6
2. BACKGROUND AND RATIONALE: 6
3. STUDY OBJECTIVES AND OUTCOMES 6
3.1. Study Objectives 6
3.1.1. Primary Objective 6
3.1.2. Secondary Objectives 6
3.2. Outcomes 7
3.2.1. Primary outcome 7
3.2.2. Secondary outcomes 7
4. STUDY DESIGN 8
4.1. Design 8
4.2. Trial Sites 8
4.3. Trial arms: 8
4.4. Randomisation and Blinding 9
4.5. Original Sample Size 9
4.6. Sample Size re-estimation .... **Hiba! A könyvjelző nem létezik.**
5. ANALYSIS POPULATIONS 10
5.1. Screening failures 10
5.2. Intention-to-treat (ITT) population 10
5.3. Per protocol (PP) population 11
5.4. Safety population .... **Hiba! A könyvjelző nem létezik.**
6. GENERAL CONSIDERATIONS FOR DATA ANALYSES 11
6.1. Reporting guidelines 11
6.2. Participant disposition and Flow chart 11
6.3. Data Summaries 11
6.4. Missing data 11
6.4.1. Endpoints 11
6.4.2. Covariates 12
6.5. Adjusted Analyses 12
6.6. Subgroup Analysis 12
6.7. Multiplicity .... **Hiba! A könyvjelző nem létezik.**
6.8. Graphical Displays 12
6.9. Software 13
7. STATISTICAL ANALYSES 13
7.1. Primary Outcome Analysis 13
7.1.1. ITT analysis of the primary outcome - the primary analysis 13
7.1.2. Covariate adjusted analysis of the primary outcome 14
7.1.3. Subgroup analysis of the primary outcome 14
7.1.4. Per-protocol analysis of the primary outcome 14
7.2. Secondary Outcome Analysis 14
7.2.1. Analysis of binary outcomes 14
7.2.2. Analysis of continuous outcomes 14
7.2.3. Analysis of count outcomes 15
7.2.4. Analysis of secondary outcomes with repeated measurements 15

7.2.5 Analysis of time-to-event outcomes 15
7.2.6 Analysis of other secondary outcomes 15
8. SAFETY ANALYSES 15
9. OTHER DOCUMENTS 15
10. REFERENCES 15
11. LIST OF SUMMARY TABLES AND FIGURES 18

ABBREVIATIONS

Abbreviation	Explanation
AE	Adverse event
BV	Bacterial vaginosis
CT	Cash transfer
ICC	Intra-Cluster Correlation
HIV	Human immunodeficiency virus
HSV-2	Human simplex virus type 2
ITT	Intention to Treat
Ksh	Kenyan Shillings
MHM	Menstrual hygiene management
SAE	Serious adverse event
SAP	Statistical Analysis Plan
SSA	Sub Saharan Africa
STIs	Sexually transmitted infections
TSC	Trial Steering Committee
VMB	Vaginal Microbiome
WASH	Water, sanitation and hygiene
95% CI	95 percent Confidence Interval

1. INTRODUCTION

The purpose of this Statistical Analysis Plan (SAP) is to define the outcome variables, statistical methods, and analysis strategies to address the study objectives in **Menstrual cups, maturation of the adolescent vaginal microbiome, and STI/HIV risk** (*Protocol version: Cups or Cash Protocol v10.1 –Jul 2021*).

2. BACKGROUND AND RATIONALE

A disproportionate number of new HIV infections occur in adolescent girls in sub-Saharan Africa (SSA) [1]. In parts of western Kenya, HIV prevalence rises from 1.3% in 13-14 year-olds to 12.8% by age 18 years [2]. Bacterial vaginosis (BV), which doubles the risk of HIV acquisition and transmission, affects 20-50% of general population women in SSA and Kenya [3-8]. For adolescent girls, the HIV/STI epidemic overlaps with broader reproductive health concerns. Menstrual hygiene management (MHM) is a pervasive problem across low- and middle-income countries and a lack of MHM materials negatively impacts girls' health and schooling. To attend school and obtain necessities such as sanitary products, soap and underwear, girls consequently often engage in exchange sex [9].

To tackle these challenges, Co-Investigator Phillips-Howard conducted a cluster randomized controlled feasibility study following 644 girls aged 14-16 years old: after one year, menstrual cup use resulted in 35% lower (p=0.034) BV prevalence and 52% lower (p=0.039) STI prevalence compared to control condition of menstrual hygiene counselling [10]. Based on our and others' research, we hypothesize menstrual cups protect against STIs by preserving or promoting a *Lactobacillus*-dominant vaginal microbiome (VMB) [11-15]. Numerous studies associated a *Lactobacillus*-dominant VMB with reduced risk of STI and HIV acquisition [16]. This study is designed to evaluate the effect of menstrual cups on the vaginal microbiome, BV, and sexually transmitted infections, among girls participating in the menstrual cups and control arms of the Cups for Cash for Girls (CCG) trial.

3. STUDY OBJECTIVES AND OUTCOMES

3.1. Study Objectives

3.1.1. Primary Objective

To determine the impact of menstrual cups, provided to secondary schoolgirls, on Bacterial vaginosis (BV).

3.1.2. Secondary Objectives

- To determine the impact of menstrual cups, provided to secondary schoolgirls, on *Lactobacillus crispatus* dominated Community State Type I (CST-I) vs. other CSTs, and relative abundance of *L. crispatus*. (Aim 1)
- To determine the impact of menstrual cups, provided to secondary schoolgirls, on composite incidence of sexually transmitted infections, *C. trachomatis, N. gonorrhoeae, T. vaginalis*. (Aim 1)
- To measure the age-specific differences in the occurrence of BV, CST-I, and STI, and risk factors for BV, non-optimal CST, and STI.

- To determine the effect of sexual debut on vaginal microbiome over time, and how this is impacted by specific sexual practices and sex partner characteristics. (Aim 2)
- If funding is available, examine the concordance of menstrual cup microbiome and vaginal microbiome, and factors (e.g., cleaning, dropping, storage) affecting this. (Aim 3)

3.2. Outcomes

Appendix 2 defines primary and secondary outcomes and data sources.

3.2.1. Primary outcome

Outcome variable BV: binary Yes/No.
Definition: Nugent score 7-10 (yes, BV) vs. Nugent score 0-6 (no BV) [17].

It is noted that

- All girls who are BV-positive at baseline can contribute to primary endpoint if they have documented antibiotic treatment.
- Those who did not have a BV test result at baseline (“unknown, refusal”), and if they agree to join for later testing and are positive, they are assumed to be ‘incident’ infections, although these could be old infections.

3.2.2. Secondary outcomes

- Nugent score: Continuous, 0-10

  Definition: Nugent score will be determined based on morphologic counts and scoring of *Lactobacillus* spp. (0-4), *Mobiluncus* spp (0-2), and gram variable rods (0-2) [17].

- Community State Type-I (CST-I): *binary: Yes/No*

  Definition: (1) participant’s VMB is classified as CST-I by VALENCIA algorithm [18]. Vs. non-CST-I VMB type (i.e., CST-III or CST-IV). CST-II and CST-V will be excluded in primary analysis given their outlier nature and limited biological characterization. In sensitivity analysis, CST-II and CST-V will be grouped with CST-I.

- Relative abundance of *L. crispatus*: continuous

  Definition: Sequence counts of *L. crispatus* will be determined by 16s rRNA sequencing of the V3-V4 regions. Relative abundance will be calculated as the percent of total sequence counts accounted for by *L. crispatus.*

- STI incidence: *binary: positive for any STI vs negative for all STIs*

Definition POSITIVE: those who are confirmed positive using Gene Xpert (Cepheid) for *C. trachomatis* or *N. gonorrhoeae*, or those who are confirmed positive using OSOM rapid diagnostic for *T. vaginalis*.

NOT POSITIVE: For those having negative test results for *C. trachomatis* AND *N. gonorrheoae* AND *T. vaginalis*, contribute as negative to the STI incidence calculation.

- Reported sexual behaviour indicators

Definition: endpoint is composed of several indicators shown below:

(i) Ever sexually active (*binary- Yes/No)*
(ii) Coercive sex (*binary- Yes/No*)
(iii) Number of sex partners (*categorical- 0, 1, 2+*)
(iv) Transactional sex (*binary – Yes/No)*
(v) Pregnancy (*binary- Yes/No*)
(vi) Condom use (*binary- Yes/No*)

Tolerability and safety endpoints

- NA for CaCHe

4. STUDY DESIGN

4.1. Design

CCG is a single site, 4-arm, open-label, matched school cluster randomised controlled superiority trial. Schools will be stratified by school size (small, medium and large according to tertile). Schools are the unit of randomisation (clusters), with girls the unit of measurement; they will be randomly allocated into 4 arms using a 1:1:1:1 ratio and permuted block randomisation (block size of 4) to minimise bias. Recruitment will be staggered over 2 school terms if logistically required. Girls will be followed-up through graduation and into employment or up to 10 academic terms to determine if they complete secondary school. Sealed, opaque envelopes will be prepared with the study allocation. Head teachers will select one sealed envelope to unveil the schools study arm during a study induction ceremony.

CaCHe is nested within CCG, with girls recruited from 6 schools randomized 1:1 – 3 randomized to menstrual cups, and 3 randomized ton control. Recruitment took place in one school term.

4.2. Trial Sites

All girls in CaCHe come from Rarieda within Siaya County.

4.3. Trial arms

CCG Participants will be randomised to one of 4 arms:

- One menstrual cup, with handwash soap termly.
- Cash transfer (CT; girls’ pocket money) Kenyan shillings (Ksh) 1500/term plus financial literacy.
- Combined menstrual cups and CT.

- 'Usual practice' control (control arm), with handwash soap termly (girls provided one cup at end of study).

All participants receive puberty and hygiene education.

CaCHe Participants come from the menstrual cup only arm or the 'Usual practice' control arm.

4.4. Randomisation and Blinding

Schools are the unit of randomisation (clusters), and girls the unit of measurement. A census of secondary schools in the area has been completed. The computer-generated randomisation list will be produced by the trial statistician in Liverpool. Schools will be randomly allocated into 4 arms using a 1:1:1:1 ratio and permuted block randomisation by school size. The actual random allocation of schools to study arms will be achieved using community ceremonies where head teachers will each simultaneously pick 1 sealed envelope from a box, with each envelope containing the allocation to one of the 4 study arms. Balanced randomisation will be achieved by ensuring that the group size of head teachers during each public ceremony will be equivalent to 1 or multiple block sizes. This allocation method was highly successful and popular in the pilot study. Because schools allocated to the 'usual practice' will receive menstrual cups at the end, cup allocation may be perceived by communities as interventions 'immediate' vs 'delayed'. The randomisation will occur prior to parent and participant meetings.

We cannot mask participants to their treatment (intervention) status due to the nature of interventions provided. However, counsellors conducting the HIV and HSV-2 testing and laboratory technicians will be blinded to the study arm. Investigators and trial statisticians will be masked. Field staff who conduct home visits to confirm dropout will also be masked when feasible. Bias will also be minimised by use of block randomisation stratified by school size at baseline. An independent person will prepare the sealed envelopes with the study allocation. Study arm allocation will not be recorded in the central database to ensure the trial statistician and data managers remain blinded throughout the study. This information will be recorded separately and only be merged with the main database following approval of the statistical analytics plan (SAP), closure of the databases and submission of a copy to the independent statistician of the Data Monitoring and Ethics Committee (DMEC).

4.5. Original Sample Size

Sample size and power calculations were performed for the minimum number of schoolgirls needed proposed 4-arm trial using NCSS/PASS, which were validated using SAS based simulation studies. Five primary comparisons of the primary endpoint were tested: (1) menstrual cup vs usual practice, (2) CT vs usual practice, (3) combined CT and cups vs usual practice, (4) combined CT and cups vs menstrual cup only, and (5) combined CT and cups vs CT only. Calculations were based on a 2-sided alpha of 0.01 to allow for 5 primary comparisons of interest, and the assumptions of an Intra-Cluster Correlation (ICC) value of 0.008 and an average secondary school target group. Taking a target of mid to late Form (class) 1 gives a sample size of an average of 46 girls, a 1 year enrolment period, a 5% overall refusal to take part in the study, 20% refusal at enrolment to consent to HSV2/HIV testing among participating girls, an average of 10 terms (~3.3 years) follow-up through the end of Form-4, and 20% loss to follow-up or refusal to provide biological samples at the end of the study period. Thus out of 46 enrolled girls/school, on average, 35 (0.95*0.80x46) will contribute to the primary analysis; 6.9 of which

will be HSV-2 or HIV positive on enrolment (24.7% of 28 girls who agree to get tested) and the remaining 28.1 will be either HSV-2/HIV negative (n=21.1) or with unknown HSV-2/HIV sero-status (n=7) because no assent/consent was provided for testing at enrolment.

A study with 4 arms of 21 schools per arm (84 schools in total) enrolling 46 girls in Form 1 per school (i.e. 966/arm; 3864 girls total) with 35 girls/school contributing to the analysis, will have 90% power to detect a 25% reduction (Relative Risk [RR]=0.75) in the 3.3-year incidence risk of the primary endpoint from 44.1% in the control group (usual practice) to 33.1% with either intervention; and 80% power to detect a 22.2% reduction (RR=0.778) to 34.3% (both at alpha=0.01). Similarly, the study has 80% power to detect a 25% difference (RR=0.75) between either intervention alone and the two in combination (from 33.1% to 24.8%) (alpha=0.025). The primary analysis of the study also has 80% power to detect a 25% reduction if the ICC is 0.0183 instead of 0.008, or the background frequency is 37.8% instead of 44.1%, or the average effective school size (after taking refusals and losses into account) is 26 instead of 35 girls.

4.6 CaCHe sample size

CaCHe was designed to estimate the effect of menstrual cups on girls' risk of BV, with an anticipated cumulative event rate of 30-40% among controls occurring over 30 months. In a design of 6 repeated measurements having AR(1) covariance structure, correlation between observations on the same subject ranging 0.25 to 0.4, and accounting for 20% loss to follow-up, group sample sizes of 220 in cup arm and 220 in control arm would achieve >80% power to detect 25% reduced prevalence of BV for the cup arm compared to control arm when BV prevalence is 30%, and 97% power when prevalence is 40% (p=0.05 two-sided test, two proportions in a repeated measures design; PASS v15 [19]).

5. ANALYSIS POPULATIONS

The analyses will be carried out by the trial statistician (Runa Bhaumik) and the primary analysis will be reviewed by a second statistician. The principle of intention-to-treat (ITT) will be the main strategy of the analysis adopted for the primary outcome and all the secondary outcomes. The membership of each analysis set will be determined and documented and the reasons for exclusion will be given prior to database lock. A summary table will list the individual subjects sorted by treatment group and describe their protocol deviation/violation.

The definitions of different analysis population are shown below:

5.1. Screening failures

If a participant gives informed assent (after parental consent) and is provided with a study ID, but then is found not to fulfil the randomisation eligibility criteria, they will be classified as a screening failure and excluded from the intention-to-treat (ITT). Pregnant girls who do not declare pregnancy at enrolment will be excluded from analysis after the dates of normal (or otherwise) deliveries confirm that the pregnancy started prior to intervention.

5.2. Intention-to-treat (ITT) population

The intention-to-treat population is defined as all participants who were from the randomised school and provided parental consent and themselves assented. These girls will be included in the intention-to-treat analysis regardless of whether they have completed all evaluations.

5.3. Per protocol (PP) population

Per protocol population will be deemed as a sub-population of the ITT population. The PP population within the menstrual cup groups is defined as all participants who report using the cup; further sub-analysis on observed change in colour showing actual use will be performed where data are available.

6. GENERAL CONSIDERATIONS FOR DATA ANALYSES

6.1. Reporting guidelines

We will follow the Consolidated Standards of Reporting Trials (CONSORT) 2010 statement; extension to cluster randomised trials guidelines for reporting of clinical trials (http://www.consort-statement.org/).

6.2. Participant disposition and Flow chart

A flow chart (e.g., Appendix 1) will be drawn up showing the number clusters allocated to each study arm and the number of girls screened, enrolled, and followed-up in each study arm, and the number contributing to the primary analysis and per-protocol.

The number screened and not enrolled and the reasons for non-enrolment will be reported, as well as the number and reasons of girls who were lost for follow up, or who were withdrawn from study for safety reason, or who crossed-over between study arms, or because of other reasons. A list of major protocol deviations will be presented after being unblindly confirmed by the Trial Steering Committee (TSC).

6.3. Data Summaries

Continuous variables will be summarised according to number of subjects with non-missing data (n), mean, standard deviation (SD), median, Q1 (lower quartile), Q3 (upper quartile), minimum, and maximum. Log transformation will be performed if deemed necessary for some variables. Categorical variables will be summarised according to the absolute frequency and percentage of subjects (%) in each category level. The denominator for the percentages is the number of subjects in the treatment arm with data available, unless noted otherwise. Event rates per 100-person years will also be reported for time-to-event clinical outcomes and adverse events of special interest.

6.4. Missing data

Missing data will be dealt with differently for the endpoint and the independent variables as follows.

6.4.1. Endpoints

Endpoints will be carefully collected. If the missing rate is too high (e.g. 20%), the priority is the quality of this study not any other remedy method. If the missing rate is low (<5%), analysis based on available data would be acceptable. Sullivan et al [20] found that multiple imputation (MI) can be inferior to complete case analysis (CCA), adding in unnecessary simulation error to missing outcome data. Therefore, in our study, the primary analysis will be based on available outcome data, which means missing data on the primary and secondary endpoints will not be imputed (8), but the missingness mechanisms will be explored and factors associated with

missingness would be adjusted in exploratory analysis. Of note, while BV and STI status may be imputed, holistic imputation of the vaginal microbiome cannot be done.

6.4.2. Covariates

If missing data is < 5%, we will conduct CCA. Else, we can opt for a multiple imputation (MI) approach based on the missing at random (MAR) assumption. Either through joint multivariate (JM; that assumes a multivariate distribution underlying the data), or fully conditional specification (FCS) which involves drawing imputations from the conditionals using Markov Chain Monte Carlo (MCMC) method. Both these methods can be applied using R or SAS. Further, imputation diagnostics will be implemented to identify anomalies in the imputed data and structure. Imputation seed will be set as 128.

6.5. Adjusted Analyses

Adjusted analyses will be performed on the primary and secondary efficacy outcomes.

The aim of the modelling is to obtain a valid estimate of an exposure-disease relationship after adjusted for confounding. The pre-specified covariables at baseline will be indicated as follows:

Cluster level

- School water, sanitation and hygiene (WASH) (score 1-5)

Individual level

- Baseline HIV status (Positive vs. not positive)
- Baseline BV status (Positive vs. not positive)
- Baseline STI status (Positive vs. not positive)
- Baseline CST-I (CST-I vs. not CST-I)
- Baseline Age (continuous)
- Baseline Ever Sexually Active (ever had sex/coerced sex vs. no)
- Measure of socio-economic status (based on the aggregate of a basket of SES indicators)

6.6. Subgroup Analysis

Subgroup analyses will be performed for the primary outcome on the ITT population. The subgroup variables will be the baseline values of: age (continuous), ever sexually active (yes vs. no), BV (positive vs. negative), CST (CST-I vs. other), and STI (positive vs. negative) as defined above for covariate adjusted analysis.

We will also explore effect modification with an interaction term between intervention and by period of study (partial COVID-19 school closure period vs. non-COVID period), by duration of intervention use (e.g. <1 vs >1y), and subgroup variables as listed above.

6.7. Graphical Displays

For binary outcomes, forest plots will be used. Kaplan-Meier plots will be produced for displaying the time-to-event data. Visualizations will also depict trajectories of specific taxa and covariates over time, stacked bar plots of taxa, non-metric multidimensional ordinations,

heatmaps, alluvial flow, and other visualizations supporting longitudinal, high dimensional analysis of microbiome data.

6.8. Software

Data management: Participant data will be collected using standardized case reporting forms on tablet computers, using ODK software. Laboratory results will be maintained in paper laboratory books and then double entered into an Excel spreadsheet or Access database (Microsoft. Redmond, Washington, USA). All data storage will be encrypted and password protected. Data Manager will extract data periodically as needed for DMEC meeting. The data will be downloaded into SAS and STATA formats for statistical analyses.

Statistical analysis: SAS (version 9.4) will be used to perform all data analyses and generate the majority of data displays. STATA or R may also be used for some data analyses.

7. STATISTICAL ANALYSES

7.1. Primary Outcome Analysis

7.1.1. ITT analysis of the primary outcome - the primary analysis

Primary outcome will be summarised as the risk of events. Generalized linear mixed models (GLMM) will be fitted to allow for the hierarchical structure of the study design. The GLMM with binomial distribution and log link function will include treatment as fixed effect, and cluster as random effect. The addition of clusters as random effect will take the matching into account that was used in the study design.

Denoting the link function of the outcome Y by log(P(Y)), the model is:

$$log\left(P\left(Y_{ijk}=1\right)\right)=\mu+\tau_k+\beta_{j(i)}+\gamma_k+\ \epsilon_{ijk}$$

Where

Y_{ijk} denotes the observed outcome value for the k-th individual from j-th school allocated to the i-th intervention.
μ is the general mean,
τ_k is the fixed effect of the intervention for k-th individual,
$\beta_{j(i)}\ random\ effect\ of\ the\ j-th\ school\ in\ the\ ith\ treatment\ arm \sim N(0,\sigma_{B^2}),$
γ_k is the covariate effect for the k-th individual.
$\epsilon_{ijk} \sim N(0,\sigma^2)$

We will generate risk ratios (RR) together with their 95% confidence intervals of having a primary endpoint for menstrual cup vs usual practice arm. In case of non-convergence, we will use a variety of approaches, such as standardizing predictors, reducing model complexity, or using a different algorithm. If the RR still cannot be estimated, the GLMM with Poisson distribution and log link function will be used. If all above models fail to converge, logistic

regression model will be used to estimate the RR as suggested by Localio, Margolis, and Berlin [21].

Testing strategy
The primary endpoint analysis will be based on the ITT population. The main conclusions for this trial will be based on the ITT analysis of the primary outcome from this model.

7.1.2. We will test one comparison (i.e., *menstrual cup vs control*) and claim statistical significance if *P* <=0.05 for the primary outcome comparison.

7.1.3. Covariate adjusted analysis of the primary outcome

To prove the robustness of the study, covariates adjusted analysis will be performed after including the pre-specified covariates listed in Section **6.5 adjusted analysis**. From this model, the adjusted point estimate and 95% CI for each comparison will be derived.

7.1.4. Subgroup analysis of the primary outcome

Subgroup analyses will be performed. We will stratify by age (≤median vs > median), sexual debut by baseline (ever had sex/coerced sex vs. no), BV by baseline (positive vs. negative), CST by baseline (CST-I vs. non- CST-I), STI by baseline (positive vs. negative).

7.1.5. Per-protocol analysis of the primary outcome
A supportive analysis of the primary outcome will also be performed among the per-protocol populations. Statistical methods will be the same as used above.

7.1.6. Sensitivity analyses of the primary outcome
Further sensitivity analyses will be used to assess the robustness of the result for primary outcome after excluding those with positive HIV, BV and STI and/or without test result at baseline. Statistical methods will be the same as used above for primary outcome

7.2. Secondary Outcome Analysis
All secondary outcomes will be analysed based on the ITT and PP population unless specified. Adjustment for multiple comparisons will consider Holm-Bonferoni method.

7.2.1. Analysis of binary outcomes
The analysis of binary outcomes will be analysed using similar crude and adjusted analysis. The same independent variables as identified in the models for the primary endpoints will be used for adjustment. Results will be expressed identical to the methods described above for the primary outcomes.

7.2.2. Analysis of continuous outcomes
The continuous outcomes (Nugent score, relative abundances; Section ***3.2 outcomes and*** **appendix 2**), will be summarised using number of subjects (n), mean, standard deviation (SD), minimum, and maximum by treatment group, and will be analysed using a GLMM linear mixed effects model with normal distribution and identity link function with treatment as fixed effect, and cluster as

random effect. Difference in mean outcome and mean differences with their two-sided 95% confidence intervals between each group will be derived from this model. If the outcome is indicated as skewed data, Wilcoxon rank-sum incorporating clustering effects will be used to test the different. Both crude and adjusted analyses will be conducted in a similar manner to the primary endpoints. Variables considered for the full models will be the same as those for the primary endpoints.

7.2.3. Analysis of count outcomes

Not applicable.

7.2.4. Analysis of secondary outcomes with repeated measurements

BV, STI, and CST-I may all be repeated measures outcomes. For repeated measure outcomes, aside from the specification stated for their relevant model, variables, such as time and interaction between treatment and time (fixed effects), baseline measurement (covariate) will need to be included to estimate the treatment effect and their two-sided 95% confidence intervals.

7.2.5 Analysis of time-to-event outcomes

For the analysis of time-to-event outcome, the Kaplan-Meier curves will be presented and compared by the log rank test by treatment group and hazard ratio and its 95% CI will be calculated using Cox regression model with the treatment as the study variable as covariate, and the cluster (i.e., school) as frailty, which will generate hazard ratios (HR) together with their 95% confidence intervals of having a primary endpoint for menstrual cup vs usual practice.

Time-to-event variables will also be summarised by the number (%) of subjects having an event and events per 100 person-years by treatment arm.

7.2.6 Analysis of other secondary outcomes

Other statistical methods may be used if deemed necessary.

7.3. Exploratory Analysis

Additional analysis which was not specified in the above will be regarded as exploratory analysis.

8. SAFETY ANALYSES

Not applicable

9. OTHER DOCUMENTS

Not applicable.

10. REFERENCES

1. Joint United Nations Programme on HIV/AIDS. *Global AIDS Update*. UNAIDS 2016. Available from http://www.unaids.org/sites/default/files/media_asset/global-AIDS-update-2016_en.pdf. Last accessed on January 31, 2017.
2. Amornkul PN, Vandenhoudt H, Nasokho P, Odhiambo F, Mwaengo D, Hightower A, Buvé A, Misore A, Vulule J, Vitek C, Glynn J, Greenberg A, Slutsker L, De Cock KM. HIV

prevalence and associated risk factors among individuals aged 13-34 years in Rural Western Kenya. *PLoS One* 2009;4(7):e6470. PMCID: PMC2714463.
3. Otieno FO, Ndivo R, Oswago S, Pals S, Chen R, Thomas T, Kunneke E, Mills LA, McLellan-Lemal E. Correlates of prevalent sexually transmitted infections among participants screened for an HIV incidence cohort study in Kisumu, Kenya. *International Journal of STD & AIDS* 2015;26(4):225–237. PMCID: PMC4709839.
4. Hemalatha R, Ramalaxmi BA, Krishnaswetha G, Kumar PU, Rao DM, Balakrishna N, Annapurna V. Cervicovaginal Inflammatory Cytokines and Sphingomyelinase in Women With and Without Bacterial Vaginosis. *Am J Med Sci* 2012;344:35-9. PMID: 22157388.
5. Mirmonsef P, Zariffard MR, Gilbert D, Makinde H, Landay AL, Spear GT. Short-Chain Fatty Acids Induce Pro-Inflammatory Cytokine Production Alone and in Combination with Toll-Like Receptor Ligands. *Am J Reprod Immunol* 2012;67:391-400. PMCID: PMC3288536.
6. Al-Harthi L, Roebuck KA, Olinger GG, Landay A, Sha BE, Hashemi FB, Spear GT. Bacterial vaginosis-associated microflora isolated from the female genital tract activates HIV-1 expression. *J Acquir Immune Defic Syndr* 1999;21:194-202. PMID: 10421242.
7. van de Wijgert JH, Morrison CS, Cornelisse PG, Munjoma M, Moncada J, Awio P, Wang J, Van der Pol B, Chipato T, Salata RA, Padian NS. Bacterial vaginosis and vaginal yeast, but not vaginal cleansing, increase HIV-1 acquisition in African women. *J Acquir Immune Defic Syndr* 2008;48:203-10. PMID: 18520679.
8. Taha TE, Hoover DR, Dallabetta GA, Kumwenda NI, Mtimavalye LA, Yang LP, Liomba GN, Broadhead RL, Chiphangwi JD, Miotti PG. Bacterial vaginosis and disturbances of vaginal flora: association with increased acquisition of HIV. *AIDS* 1998;12:1699-1706. PMID: 9764791.
9. Phillips-Howard PA, Otieno G, Burmen B, Otieno F, Odongo F, Odour C, Nyothach E, Amek N, Zielinski-Gutierrez E, Odhiambo F, Zeh C, Kwaro D, Mills LA, Laserson KF. Menstrual needs and associations with sexual and reproductive risks in rural Kenyan females: A cross-sectional behavioral survey linked with HIV prevalence. *J Womens Health (Larchmt)* 2015;24:801-11. PMCID: PMC4624246.
10. Phillips-Howard P, Nyothach E, ter Kuile F, et al. Can menstrual products improve African girls' school attrition, and sexual and reproductive health? Evidence from a cluster randomised controlled feasibility study, rural western Kenya. *BMJ Open* 2016; 6:e013229.
11. North B, Oldham M. Preclinical, clinical, and over-the-counter postmarketing experience with a new vaginal cup: menstrual collection. *J Womens Health* 2011;20(2):303-311. PMCID: PMC3036176
12. Beksinska ME, Smit J, Greener R, Todd CS, Lee ML, Maphumulo V, Hoffmann V. Acceptability and performance of the menstrual cup in South Africa: a randomized crossover trial comparing the menstrual cup to tampons or sanitary pads. *J Womens Health (Larchmt)* 2015; 24(2):151-8.
13. Sommer M. Where the education system and women's bodies collide: The social and health impact of girls' experiences of menstruation and schooling in Tanzania. *Am J Public Health* 2015;105(7):1302-11.
14. Anand E, Singh J, Unisa S. Menstrual hygiene practices and its association with reproductive tract infections and abnormal vaginal discharge among women in India. *Sexual & Reproductive Health Care* 2015;6(4):249-254.

15. Das P, Baker KK, Dutta A, Swain T, Sahoo S, Das BS, Panda B, Nayak A, Bara M, Bilung B, Mishra PR, Panigrahi P, Cairncross S, Torondel B. Menstrual hygiene practices, WASH access and the risk of urogenital infection in women from Odisha, India. *PLoS One* 2015;10(6):e0130777. PMCID: PMC4488331.
16. van de Wijgert JH, Borgdorff H, Verhelst R, Crucitti T, Francis S, Verstraelen H, Jespers V. The vaginal microbiota: what have we learned after a decade of molecular characterization? *PLoS One* 2014;9(8):e105998. PMCID: PMC4141851.
17. Nugent RP, Krohn MA, Hillier SL. Reliability of diagnosing bacterial vaginosis is improved by a standardized method of gram stain interpretation. *J Clin Microbiol* 1991;29:297-301.
18. France MT, Ma B, Gajer P, Brown S, Humphrys MS, Holm JB, Waetjen LE, Brotman RM, Ravel J. VALENCIA: a nearest centroid classification method for vaginal microbial communities based on composition. *Microbiome* 2020;8:166. PMCID: PMC7684964.
19. Hintze J. (2014). PASS 15. NCSS, LLC. Kaysville, Utah, USA. URL *http://www.ncss.com*
20. Sullivan TR, White IR, Salter AB, Ryan P, Lee KJ. Should multiple imputation be the method of choice for handling missing data in randomized trials? *Stat Methods Med Res* 2018; **27**(9): 2610-26.
21. Localio AR, Margolis DJ, Berlin JA. Relative risks and confidence intervals were easily computed indirectly from multivariable logistic regression. *J Clin Epidemiol* 2007 Sep;60(9):874-82.

11. LIST OF SUMMARY TABLES AND FIGURES

**Section 1**	**Disposition of subjects**
Table 1.1	Subjects recruitment and disposition by cluster
Table 1.2	Subjects recruitment and disposition by treatment
Table 1.3	Summary of protocol deviation/violation form
Table 1.4	Summary of follow up time
Table 1.5	List of subject excluded from ITT, PP and safety population
**Section 2**	**Baseline characteristics of subjects**
Table 2.1	Enrolment information (ITT analysis)
Table 2.2	Lab tests at baseline (ITT analysis)
Table 2.3	Sexual exposure at baseline survey (ITT analysis)
Table 2.4	Menstruation at baseline (ITT analysis)
**Section 3**	**Follow-up results**
Table 3.1	Summary of Sexual exposure by follow-up visit (ITT analysis)
Table 3.2	Summary of Menstruation by follow-up visit (ITT analysis)
Table 3.3	Summary of lab tests by follow-up visit (ITT analysis)
**Section 4**	**Analysis of primary outcome**
Table 4.1	Number (%) of primary outcome by treatment (ITT analysis)
Table 4.1a	Number (%) of primary outcome by treatment (PP analysis)
Table 4.2	Crude analysis of primary outcome (ITT analysis)
Table 4.2a	Crude analysis of primary outcome (PP analysis)
Table 4.3	Adjusted analysis of primary outcome (ITT analysis)
Table 4.3a	Adjusted analysis of primary outcome (PP analysis)
Table 4.4	Subgroup analysis of primary outcome (ITT analysis)
**Section 5**	**Analysis of secondary outcome**
Table 5.1	Summary of secondary outcome by treatment (ITT analysis)
Table 5.2	Crude analysis of binary secondary outcome (ITT analysis)
Table 5.3	Crude analysis of time-to-event secondary outcome (ITT analysis)
**Section 6**	**Analysis of safety outcome**
NA	
**Figures**	
Figure 7.1	Number (%) of primary outcome by treatment and visit

Appendix 1

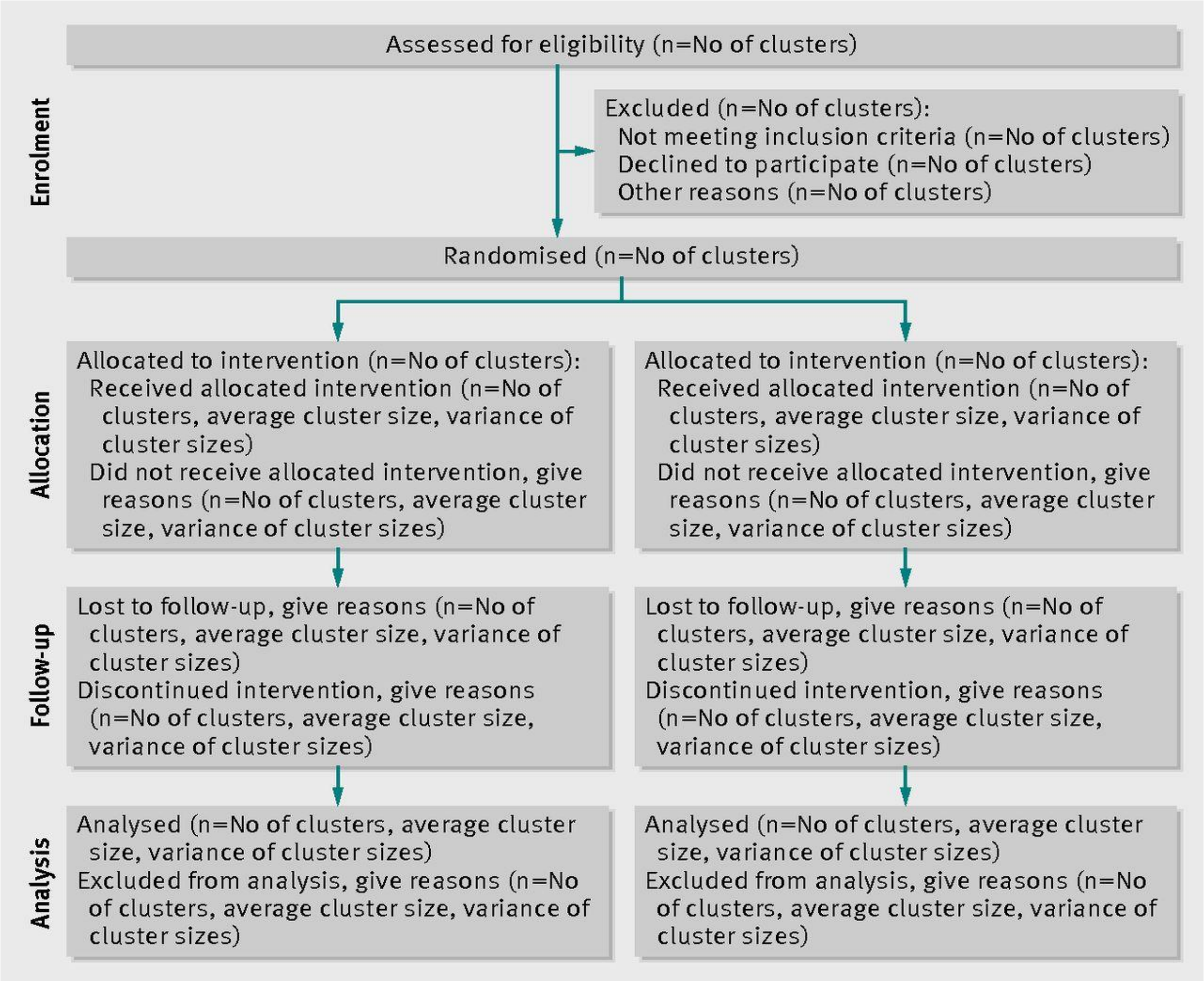
Enrolment
Assessed for eligibility (n=No of clusters)
Excluded (n=No of clusters):
Not meeting inclusion criteria (n=No of clusters)
Declined to participate (n=No of clusters)
Other reasons (n=No of clusters)
Randomised (n=No of clusters)
Allocation
Allocated to intervention (n=No of clusters):
Received allocated intervention (n=No of clusters, average cluster size, variance of cluster sizes)
Did not receive allocated intervention, give reasons (n=No of clusters, average cluster size, variance of cluster sizes)
Allocated to intervention (n=No of clusters):
Received allocated intervention (n=No of clusters, average cluster size, variance of cluster sizes)
Did not receive allocated intervention, give reasons (n=No of clusters, average cluster size, variance of cluster sizes)
Follow-up
Lost to follow-up, give reasons (n=No of clusters, average cluster size, variance of cluster sizes)
Discontinued intervention, give reasons (n=No of clusters, average cluster size, variance of cluster sizes)
Lost to follow-up, give reasons (n=No of clusters, average cluster size, variance of cluster sizes)
Discontinued intervention, give reasons (n=No of clusters, average cluster size, variance of cluster sizes)
Analysis
Analysed (n=No of clusters, average cluster size, variance of cluster sizes)
Excluded from analysis, give reasons (n=No of clusters, average cluster size, variance of cluster sizes)
Analysed (n=No of clusters, average cluster size, variance of cluster sizes)
Excluded from analysis, give reasons (n=No of clusters, average cluster size, variance of cluster sizes)

Appendix 2 Pre-specified outcomes in CaCHe study

Outcomes	Definition	Time points (analysis)	Questions in CRF/Data Source	Type of data
**Primary outcome**				
Incident BV	Nugent score 7-10 vs. 0-6	Measured Every 6 months	Derived from each individual laboratory record	Binary
**Secondary outcomes**				
Nugent score	0-10	Measured every 6 months	Derived from each individual laboratory record	Continuous
CST-I	Microbiome composition classified by VALENCIA algorithm	Measured Every 6 months	Derived from each individual laboratory record	Binary
Relative abundance of *L. crispatus*	Proportional abundance of *L. crispatus* relative to total read count	Measured Every 6 Months	Derived from each individual laboratory record	Continuous
STI incidence	For those who were confirmed by STI laboratory test testing	baseline, midline and end of study	STI status on laboratory report form	Binary/time-to-event
**Aim 2**				
**Reported sexual behaviour indicators**				

(i) Sexually active		Measured Every 6 months	(a) Have you ever had sex with a man or boy? (b) Has a man or boy ever forced or threatened you to make you have sex?	Binary
(ii) Coercive sex		baseline, midline and end of study	(a) The first time you had sex (or were forced or threatened to make you have sex with) a boy or man, did you want to have sex with this man or boy? (b) In the past 6 months, have you been forced or threatened to make you have sex with a man or boy? (c) In the past 30 days, have you had sex (or been forced or threatened to have sex)?	Binary
(iii) Transactional sex		baseline, midline and end of study	(a) If you have ever had sex or been forced by a man or a boy to have sex, why did it first happen? (offered money, given a gift/favour); (b) Does the man or boy give you something for having sex with him?; (c) Who has been the main person that provided you with pads (or money for pads)? (Person giving money after sex, Person giving pads after sex); (d) Now tick any of the persons that have ever (baseline; past 12 months follow-up) given you pads (or money for pads)? (Person giving money after sex, Person giving pads after sex); (e) During your most recent period, did you have to do things to get pads or other menstrual items? If yes, what? (Sex); (f) Apart from schoolwork, do you have other tasks you must do? Tick any that you did in the last month. (Sex work); (g) Have you ever had sex in order to pay for things, or get favors?; (h) In the last month did anyone give you money, gifts, or presents? If yes, why did they give you the money/gift/present? (After sex); (i) If you get money from a man or boyfriend do they request sex in return?	Binary
(iv) Number of sex partners		baseline, midline and end of study	(a)How many boys or men have you had sex with (or been forced or threatened to make you have sex with) **in your life**? (b) In the past 6 months, how many boys or men have you had sex with (or been forced or threatened to make you have sex with)?	Categorical

(v) Pregnancy		baseline, midline and end of study	(a) Are you currently pregnant? (b) Have you ever been pregnant? (c) How many times have you been pregnant?	Binary/Categorical
(vi) Condom use		baseline, midline and end of study	(a) have you or your partner ever used condoms to delay or avoid a pregnancy or sexually transmitted infection? (b) In the last 6 months, have you or your partner used condoms to delay or avoid a pregnancy or sexually transmitted infection? (c) In the past 6 months, how often did the boy or man you were having sex with (or forced or threatened to make you have sex with) use a condom? (categorical (ordinal): never(0), rarely (1), sometimes (2), often (3), always (4)) (d) The last time you had sex (or were forced or threatened to have sex), did the boy or man use a condom? (categorical: Yes/No)	Binary/Categorical
**Microbiome Indicators**				
(i) CST		Measured Every 6 Months	Community state type (CST) as defined by VALENCIA algorithm	Categorical
(ii) VMB		Measured Every 6 Months	Relative abundance of taxa	Continuous
