# Supplementary material for: Analysis of bacterial vaginosis, the vaginal microbiome, and sexually transmitted infections following the provision of menstrual cups in Kenyan schools: Results of a nested study within a cluster randomized controlled trial
Source: PLoS Med. 2023 Jul 25;20(7):e1004258. doi: 10.1371/journal.pmed.1004258 (PMC10368270; doi:10.1371/journal.pmed.1004258)
Supplement: Attachment — Submitted filename: CaCHe rebuttal responses 5Feb23.docx [file pmed.1004258.s006.docx]

**Response to Editors’ note received on 30Jan23**

* As well as the proposed changes you provided in your rebuttal letter/tracked manuscript, please also ensure that the study design is clearly described to minimise the risk of further misunderstanding by readers. Clarifications can be guided by your replies to Reviewer #2.

- **We have added explanation of why the nested study was carried out as observational; specifically, that it would have been infeasible, unethical, and statistically unnecessary to re-randomize within the randomized cluster.**

* Related to the above, please ensure the observational nature of your prospective cohort study is reflect in the language use (avoid causal language such as 'effect', including in the title). Refer instead to associations.

- **We have made edits in the title, abstract, and text.**

* Given that your study is a cohort study nested within an RCT, please complete a STROBE checklist in your Supporting Information and cite this in the Methods. Please use section headings and paragraph numbers (rather than line/page numbers) when completing the form.

- **We have completed the STROBE checklist (using section headings and paragraph numbers) and submitted as Supporting Information, with call in the Methods under the section *Study Setting, Design, and Participants*.**

* In the final sentence of your Abstract Methods and Findings, please describe the study limitations.

- **We have summarized primary limitations in the final sentence of Abstract Methods and Findings.**

* Please remove the Funding Source and Role.

- **We have removed this from the text.**

* Please include an Author Summary.

**🡪 We have added an Author Summary. Since the whole section was new, we do not put it in track changes.**

* Please update citations to be normal script, preceding punctuation, and placed within square brackets.

- **We have done this.**

* Please cite Supporting Information per our guidelines.

- **We have done this.**

* Please remove the 'Role of the funding source' section.

- **We have done this.**

* Please remove the 'Author Contributions' section.

- **We have done this.**

* Please remove the Declaration of Interests' section.

- **We have done this.**

* Please ensure that any changes to the planned analyses conducted in response to peer review comments are clearly identified as such in the Methods.

- **There were no changes in response to the planned analyses in response to reviewers. The additional “digging” on specific confounding for Reviewer 3 was informational to answer their curiosity only and is not reported in the paper.**

* Please define loss to follow up, as used in this study, in the Methods.

- **We have added definition of loss to follow up and clarified this as missed visit rather than completely lost to follow-up; participants with no follow-up visits are summarized in the preceding sentence. (“Lost to follow-up” follows the CONSORT flow diagram reporting terminology, which includes missed visits.)**

  * In the Discussion, please also discuss generalisability, highlighting that in the trial setting BV (Nugent score >7) was treated, and screening and treatment for BV is not usually done.
- **We have done this.**

****In addition to these edits and those made in response to the reviewers below, we clarify in Table 1 the type of pads used (the survey question did not differentiate disposable from reusable pads – it asked about sanitary pads, and we have made this correction.)**

**Reviewer #1: Statistical review**

This paper reports a substudy of a cluster randomised trial that examines whether menstrual cups reduce the prevalence of bacterial vaginosis. The authors demonstrate that there is a significant reduction in the primary analysis.

Generally the study used appropriate methods and was reported well. I have some comments below:

- **Thank you for your positive review and helpful comments. We have made the clarifications as detailed below.**

  1.      Abstract + results page 14: I would recommend providing the p-values for the secondary outcomes in addition to the CIs.
- **We do not report p-values for secondary outcomes because we were *a priori* powered only for the primary outcome (BV), and the study design and the SAP did not specify multiple comparison adjustment** [Harrington et al.]**. We have clarified in the statistical analysis section that study design and SAP did not pre-specify multiple hypothesis testing and therefore p-values are presented only for the primary outcome.**
- Harrington D, D’Agostino RB, Gatsonis C, Hogan JW. New guidelines for statistical reporting in the journal. *N Engl J Med* 2019;381:285-286.

2.      Page 8, sample size calculation: is the 25% reduced prevalence in absolute terms (i.e. from 30% to 5%) or in relative terms? Was any clustering effect allowed for?

- **We have added the information that this was a relative difference.**
- **Clustering was accounted in the CCG study and not in this nested study. Had we included clustering effect in the sample size calculation for this nested study, a larger sample size would have been indicated. Our estimates for 80% power to detect 25% relative difference in primary outcome of BV were based on an expected 30% cumulative prevalence, but in our findings it was 39%.**

3.      Statistical methods: the follow-up rate was generally quite high, but it would be good to mention what assumptions about missing data were made (i.e. that the analysis model allowed for missing at random patterns of missingness).

- **We have added this clarification.**

4. Statistical methods: can the authors provide some intuition about how to interpret the estimated odds ratio - is this basically an average across all time points?

- **We have added this clarification.**

5.      Page 14: I feel that the text should point out that the multivariable adjusted model for the primary outcome no longer was significant (especially as the authors highlight a secondary endpoint becoming significant with the adjustment).

- **This is a fair point, and this was raised by another reviewer. In the results we have added, “, though the association between cup arm and BV was no longer statistically significant” regarding adjusted analyses for BV.**

James Wason

**Reviewer #2:**

This manuscript presents the results of a cohort study nested within a randomized trial examining the effect of menstrual cups compared to usual care on bacterial vaginosis in a population of adolescent girls and young women.  The authors conclude that menstrual cups benefitted adolescents by reducing BV and increasing lactobacillus crispatus relative abundance.  There are some critical analytical problems that lead me to seriously question the primary findings.  In addition, the authors discuss the findings as though they are derived from a RCT.  The net effect is that they make strong statements about the benefits of the menstrual cup based on findings that do not seem justified by the data presented.

Major Comments
1)      Methods state that "All girls with Nugent score 7-10 were treated due to lack of correlation of reported symptoms with BV."  This rationale does not justify treating asymptomatic BV - the only indication for treatment of asymptomatic BV is in women who are pregnant and have a history of preterm birth.  Even this indication is currently being questioned.  Equally importantly, the decision to treat all BV in this cohort means that the comparison groups were actually menstrual cup plus regular screening and treatment of BV versus regular screening and treatment of BV.  It does not really address the question of whether menstrual cup alone reduces BV in a realistic setting where only symptomatic BV is treated.

- **Regarding treatment of BV and STIs: It would have been unethical to screen for a disease in this setting with extremely limited access to healthcare and not provide treatment, and the entire team - Kenyan, US, UK (including clinician investigators and staff clinicians) - supported this decision as did the ethical review committees overseeing the study.**
- **Regarding whether menstrual cup alone reduces BV: Both arms were tested and treated equivalently for lab-detected BV and STIs, therefore we did not introduce a study bias or create a differential effect by study arm.**

2)      The results state that baseline BV, STI, and CST were similar between groups.  This is not true for BV, the primary outcome of the trial.  In fact, the BV prevalence in the control arm was 13.5% (30/223) while the prevalence of BV was 8.9% (19/213) in the cup arm.  By a quick calculation, this appears to yield an odds ratio of 0.66 for BV in the cup versus control arm prior to the intervention - bigger than the effect seen later in the study.  Since the authors describe this as a cohort study, it seems like they should adjust for this baseline difference in the primary outcome variable.  Instead, the analysis was conducted as though this was an RCT.  Even if you consider it to be a small cluster RCT with too few clusters, it would be important to adjust for a baseline difference if the randomization did not result in similar rates of the outcome in the two arms.

- **Cups or Cash for Girls (CCG) was a cluster randomized controlled trial. This current study, CaCHe, is nested within that. Because CCG was randomized, with participants in 2 of the 4 arms included in CaCHe, it would not have been feasible, ethical, or statistically necessary to again randomize, when already randomized. This does not negate the randomization, which was fully described by Zulaika et al 2019, which is cited.**
- **The adjusted analyses are presented alongside the crude in the primary results table. The variables adjusted for include age, WASH, and SES. BV at baseline is included in the baseline stream. The effects sizes remain similar in crude and adjusted analyses, showing that association between treatment arm and BV was independent of these baseline differences.**

3) Discussion states, "Our findings build on these studies, showing that menstrual cup use can benefit the vaginal microbiome and reduce BV, measured using sensitive molecular methods, in the setting of a rigorous cluster randomized design."  This is misleading - the analysis presented in this paper is a cohort (or a cRCT with too few clusters), and did not account for an important baseline difference in the outcome.

**🡪 As noted above, we do account for important baseline differences, and the study is randomized. As stated above, since our 2 conditions of interest (control, and cups alone) were already randomized in the CCG, we do not re-randomize for CaCHe, thus this is a nested cohort, and we observe the randomized subjects.**

**Reviewer #3:**

Mehta and colleagues used data from a cluster randomized trial conducted among secondary schools in Kenya to evaluate the effect of menstrual cup use, versus normal menstrual management practices, on BV, the vaginal microbiome, and STI. Their data indicate that individuals attending schools randomized to receive menstrual cups had 24% reduced odds of BV and 37% higher odds of L. crispatus-dominated microbiota. These novel findings warrant replication in additional populations, but they suggest provision/promotion of menstrual cups may be a relatively easy-to-implement intervention to improve vaginal microbiota composition and reduce BV. Overall, the manuscript is well written and clear. I'll share some suggestions with the goal of improving reader experience and the dissemination of these important and exciting findings.

**🡪 Thank you for the thoughtful review, supportive comments, and constructive suggestions to improve reporting and readability.**

Major comments
1.      In the Methods, please provide details on Gram stain and Nugent score quality control and quality assurance measures. With BV assessed by Nugent score as the primary outcome, understanding the efforts taken to reduce inter-observer variation and improve accuracy in Nugent scoring is essential to evaluating the validity of the findings.

**🡪 We have added this. All slides were read by two technicians, and where they disagreed, they reviewed the slide until they came to consensus.**

2.      In the Methods, antibiotic-use-related inclusion and exclusion criteria are unclear and presented/described differently in several places (lines 164-165, 193-196, 211-214). The rationale for these criteria is also unclear. Please more clearly describe the criteria and why they were used (I think to allow for repeat outcomes following antibiotic-mediated BV/STI clearance?), and please include all relevant information together in one place.

**🡪 Reviewer is correct! The clarification has been done – thank you.**

3. A figure depicting participants' baseline and end-of-follow-up microbiota composition (e.g. stacked bar plot, heatmap), indicating whether participants attended schools in the menstrual cup versus control arm, and indicating presence of BV and STI would be very helpful in conveying the microbial context of the trial and its findings. Please consider including such a figure.
**🡪 We agree this enhances the information for reviewers and have added a supplemental figure of two stacked bar charts (separately for all control observations and all intervention observations) showing distribution of taxa with highest mean relative abundance, according to community state type, with presence of BV and STI status indicated.**

4. The first paragraph of the Results seems to undersell/under-report some of the differences in baseline characteristics between the intervention arms. It is somewhat subjective what constitutes a small vs. large difference, but here are some comments from the text I'd suggest reconsidering/revising:

a.      "some imbalance in…school WASH score" (line 247) implies minor differences. However, about half of menstrual cup schools had a score of 0 compared to about a quarter of control arm schools.

- **This is fair and we have revised the statement to: “Baseline characteristics were similar by study arm in relation to age, SES score, and sexual activity (Table 1; N=223 control arm, N=213 menstrual cup arm), but with large imbalance in WASH scores among participants in the control schools.”**

b.      "Baseline BV…were similar" (line 248). Similar proportions of participants had Nugent score 0-3 at baseline; however, the prevalence of Nugent score 4-6 was two-fold higher in menstrual cup schools, and the prevalence of Nugent score 7-10 was about 1.5-fold higher in control arm schools.

- **While we refrain from presenting *post-hoc* p-values on the baseline characteristics, this difference is substantial, but not statistically significantly different at the p<0.05 level. Importantly, baseline BV status is adjusted for in the longitudinal data stream.**

c.      Among ever-sexually-active participants, condomless sex/never using condoms was 2-fold higher among menstrual cup schools. This may be worth mentioning in the text.

- **Because condom use was a subset of those who reported being sexually active (which was unfortunately subject to substantial underreporting [Mehta et al. *Front Cell Infect Microbiol* 2021; https://doi.org/10.3389/fcimb.2021.716537]), we present these sexual practice details only informationally for readers, and have noted this as a limitation (lines 389-391).**

5.      I think the subgroup analysis stratified by history of transactional sex deserves more attention in the discussion (because its exciting)! The BV OR was substantially lower in magnitude among those reporting a history of transactional sex than among those reporting never engaging in transactional sex. Neither OR is significant, and the CI for those reporting transactional sex is wide, so it will be important to not over interpret these findings. That said, this difference and the data suggesting a potentially stronger protective effect among those who do report history of transactional sex directly speak to one of the hypothesized sociobehavioral mechanisms by which menstrual cup use may reduce BV and improve vaginal health - that menstrual cups eliminate (at least one) cause/motivation to engage in transactional sex, which is to be able to afford/acquire menstrual management materials.

- **We appreciate your consideration and interest. However, we refrain from too much interpretation on the sub-group analyses due to limited sample sizes and resultant extremely wide 95% CI. We note this in the limitations, as to power for interaction would have been infeasible.**
- **As an aside, we note that a subsequent R01 being led by Mehta will be assessing the effects of menstrual cups on VMB, BV, and STIs among sex workers.**

6.      Not reporting data on either baseline menstrual management practices or follow-up adherence to menstrual cup use among those attending menstrual cup schools are important limitations that should be discussed. Alternatively, if these data are available, please include them in the descriptive statistics.

🡪 **We apologize for this oversight and have added this data to Table 1.**

Minor comments
1.      The hypothesis that menstrual cup use may reduce reliance on transactional sex to afford/acquire menstrual management materials (lines 62-63) should be explicitly stated earlier in the introduction.

- **We removed the hypothesis on reliance on transactional sex, as that wasn’t a hypothesis of the current study (rather was folded in from CCG).**

  2.      The language used to describe socioeconomic status categories (line 110) is stigmatizing, especially considering you refer to both categories as "poor." I would recommend using more a quantitative description (e.g. just referring to the cutpoint between quintiles 2 and 3). Please also be sure to use the same language to describe these categories in the Methods, Table 1, and Figure 3.
- **We have changed this to lowest quintiles description in the text and clarified the data labels in Table 1.**

  3.      Please clarify whether the "water for handwashing" component of the WASH score (line 112) refers to clean water or any water.
- **This refers to any water, and have clarified this in the text. It was not feasible to capture the source of water nor measure its quality (e.g., colliform bacteria, turbidity), and** **the water used may have been from more than one source.**

  4.      Throughout the methods, please provide the version number for all software used.
- **We note for Pass (v15), SAS (v9.3), Stata (v17), and R (4.1.3).**

  5.      Please describe the design of study follow-up in the text of the Methods (e.g. the number and timing of follow-up visits).
- **We apologize for this oversight and have added this.**

  6.      In lines 153-155, the order and timing of which institutes received and processed swabs for BV and STI detection is unclear. Please revise.
- **We have re-ordered the sentences and more clearly specified the which swabs were processed where and when.**

  7.      Please state the cutpoints/strata for subgroup analyses in the Methods (lines 224-226).
- **Given sparsity and word limit constraints, we rely on the variable labels in the Figure which specify all the cut points. However, in revising, we found an error on the variable labels for SES and have corrected this.**

  8.      Results paragraph in lines 271-277 should refer to Table 3
- **We have double checked and believe these are in Table 2.**

  9.      There are a few places that would benefit from additional references:
  a.      Line 324 - more than one reference should be provided as microbiota destabilization during menses is, as you state, well documented.
- **We have added references here.**

b.       Line 327 - please provide a reference(s) for iron being favorable to Gardnerella and a primary/original research reference for Gardnerella being a major BV pathobiont (not just a review).

- **We have added to and updated our references.**

c.      Lines 342-345 - again, please provide primary/original research reference(s) for poor quality menstrual management materials being associated with adverse outcomes.

- **Because the citation by van Eijk et al. (*Lancet Public Health* 2019) is a meta-analysis of original research we do not add citations here.**

10.     It is important to note that these findings may not be generalizable to individuals attending secondary school in regions other than East Africa, sub-Saharan Africa, especially considering typical menstrual management practices are often "passed down" matrilineally and likely differ between global regions.

- **This is a good point and we have added it to the Limitations “…results for those who are not in school or in different global regions are unknown.”**

  11.     Please place the menstrual cup arm and control arm columns in the same order in Table 1, Table 2, and Supplemental Table 2.
- **Thank you, we have done this.**

  12.     In Figure 3, please include axis tick marks and labels for ORs <1. Please also clarify in the caption whether a non-linear scale has been applied to the x axis.
- **Apologies for the oversight. We have added tick marks and labels for the ORs <1.**
- **The graph is on the log scale and we have added this information to the figure legend.**

  Signed: Kayla A. Carter

**Reviewer #4:**

This is a well-conducted study on an important topic to improve vaginal health in adolescent girls and young women in a setting with high HIV and STI burden. The study appears to be rigorous in its rationale, design, and analysis. I have only minor revisions and suggestions regarding the analysis.

**Thank you for your thoughtful review and supportive comments! We appreciate the suggestions to improve readability and clarity.**

1.      Is it worth carrying out another sensitivity analysis that leaves out BV-intermediate? Lumping BV intermediate in with BV negative could be diluting the effects, especially given the L. crispatus finding is statistically stronger. Perhaps BV negative could be compared to BV positive, or BV positive and intermediate together to BV negative. A lot of the BV intermediate could be L.iners dominated.

- **Reviewer is most likely correct that this would strengthen results for reasons described above. Also, as seen in the newly added Supplemental Figure 1 – the most stark contrast in BV and STI outcomes is between CST-I (*L. crispatus* dominated) and CST-IV (diverse; where most BV and STI outcomes occur). However, this isn’t in the statistical analysis plan, and we refrain from over-interrogation of primary outcome.**
- **As an aside, Specific Aim 2 of the grant will account for time-varying co-variates to identify demographic and sexual practice factors associated with transitioning between community state types.**

2. There is analysis of the absolute abundance of L.crispatus. It would be of interest to know what the menstrual cup is doing to overall bacterial load, in addition to relative abdunance.

- **We didn’t measure bacterial load and we have added this as a limitation.**

3. Should relative abundance of L.crispatus vs L.iners vs non-lacto be considered?

- **Reviewer suggests a reasonable comparison, given the three dominant community state types we observed and the biological relevance of each. However, we were not designed/powered to make this comparison and the *a priori* SAP sought to examine optimal (CST-I) vs. non-optimal and relative abundance of *L. crispatus*. However, as with Reviewer’s previous point above, it’s likely that results would be *strengthened* if we accounted for *L. iners* (CST-III/intermediate) separately from CST-IV (non-optimal/most likely to be associated with BV).**

4. The authors could specify the number and intervals of visits in the design. At the end of the methods and in the figures, this appears to be 6-monthly, but the number of observations per participant is an important aspect of the study.

- **Apologies for the oversight; we’ve added this to the Methods.**

5. The difference in unadjusted vs adjusted STI effect is puzzling. Why do the 95% CI vary so widely (wide in unadjusted but narrower in adjusted, with a similar effect size?) Similarly, the 95% CI for the BV effect are narrow in unadjusted but quite wide in the adjusted, despite similar effect sizes for menstrual cups vs BV - it would be useful to know what the key variables are that are changing these outcomes so dramatically.

- **BV and STI coefficients do not change dramatically when multivariable adjusted:**
  - **BV: 0.76 crude 🡪 0.82 adjusted (7.9% change)**
  - **STI: 0.82 crude 🡪 0.77 adjusted (6.1% change)**
- **Therefore, we don’t want to speculate on these changes in the text.**
- **However, for reviewer’s interest:**
  - **BV: If we look at the OR adjusted for each of the *a priori* specified confounding variables one at a time, none of them changes more than 10%: 0.74 (sexactive), 0.77 (SES), 0.78 (WASH), 0.80 (age).**
    - **The adjusted model loses precision primarily when adjusted for age and for sexually active (both of which are associated with BV, but not with randomization arm). Taken together, while there is not evidence of confounding, there is loss of precision resulting in wider confidence intervals that include 1, corresponding to p-value >0.05.**
  - **STI: If we look at the RR adjusted for each of the confounders one at a time there is minimal change: 0.84 (age), 0.82 (SES), 0.78 (sexactive). The biggest change comes from WASH (0.73; 11% change). The confounding with WASH stems from the baseline imbalance by group, and because WASH score is associated with reduced STI for both control and intervention participants. (note, WASH score was not associated with BV)**
- **Regarding why STI *becomes* significant, there are two potential explanations: (1) Adding a significant regressor (WASH) reduces the error variance; in doing so, other regressors may become more significant even if the parameter estimate remains the same. (2) If there is dependence between regressors (as noted, WASH score was imbalanced by group assignment), this can alter coefficients and significance.**
- **As the crude effect of menstrual cups on STI was not significant, we do not highlight this in the Conclusions of the paper. The results need to be replicated – especially in populations who have varying sexual exposures.**

6.      Were any interaction analyses considered for Fig3? Even if non-significant these could be worth reporting, given some of the differences between strata.

- **No tests of interaction were conducted due to small n per strata**
- **Most of these point estimates are generally similar, except for STI positive at baseline (positive OR for positive and inverse OR for STI negative), SES (inverse OR for lower and ~1 for higher), median age (inverse OR for above median; ~1 for younger age), and transactional sex (inverse OR for transactional sex; ~1 for no transactional sex).**
- **We refrain from drawing any inference on this given the very wide and substantially overlapping confidence intervals.**

7.      There is no comment in the discussion on cost-effectiveness versus effect size- do these results favour scaling up this intervention? Would the increasing availability of reusable cups favour their widespread distribution? While its clearly beyond scope to do this analysis here, some speculation re: future directions would be useful.

- **Phillips-Howard’s recently completed CEA among schoolgirls in Kenya demonstrates the cost-effectiveness of cups compared to other MHM approaches in the current setting, with DALYs accounting for impacts on STIs and reproductive tract infections. We have added this citation.**
- **Increasing cup availability (distribution and price point) would certainly support uptake. We hope that results of studies such as ours contribute to increased demand and distribution.**
