# Supplementary material for: Analysis of bacterial vaginosis, the vaginal microbiome, and sexually transmitted infections following the provision of menstrual cups in Kenyan schools: Results of a nested study within a cluster randomized controlled trial
Source: PLoS Med. 2023 Jul 25;20(7):e1004258. doi: 10.1371/journal.pmed.1004258 (PMC10368270; doi:10.1371/journal.pmed.1004258)
Supplement: Attachment — Submitted filename: CaCHe rebuttal responses 31Mar23.docx [file pmed.1004258.s007.docx]

**Comments from the Academic Editor:**

1. Change in title to better reflect study design.

- **Following advice from editors and to prevent confusion with observational study, we have clarified the study design as “Results of a nested analysis within a cluster randomized controlled trial”. We have made similar revision in the language of the text throughout the manuscript.**
- **Given editor advice and Reviewer 2 acknowledgement of the trial design, we have reverted to the CONSORT checklist and added the CONSERVE-CONSORT extension checklist, and removed the STROBE checklist that was submitted with the first revision. We have also reverted description of findings in a trial context (i.e., effect rather than association).**
- **We have also included the Statistical Analysis Plan as supplementary material.**

  2. Description of the intervention as cup plus screening and treatment of BV versus screening and treatment of BV.
- **This has been added on Page 8, paragraph 1, “The cup and control participants under CaCHe differed from CCG in that they all received BV and STI testing and treatment, thus the comparison was cups plus BV and STI testing and treatment vs. control plus BV and STI testing and treatment.”**

3. Clear statement regarding generalisability of the findings in the discussion.

- **We have added the following (underline) to the Discussion: “Though both arms were tested and treated equivalently for BV and STIs, we treated BV (Nugent score 7-10) regardless of symptoms due to lack of correlation with diagnosis [16],** **and this is not standard of care for BV, and therefore results may not generalize to those not screened or treated for BV.”**

**Requests from the editors:**

1. Please respond to the comments from Reviewers 2 and 3.

- **Point by point responses to the additional comments from Reviewers 2 and 3 are provided below.**

2. Did your sub-study have its own associated prospective protocol or analysis plan (separate from the trial protocol available at <https://nam04.safelinks.protection.outlook.com/?url=https%3A%2F%2Fbmcpublichealth.biomedcentral.com%2Farticles%2F10.1186%2Fs12889-019-7594-3&data=05%7C01%7Csupriyad%40uic.edu%7Cf43910907731429b48c308db248ab24b%7Ce202cd477a564baa99e3e3b71a7c77dd%7C0%7C0%7C638143950350274163%7CUnknown%7CTWFpbGZsb3d8eyJWIjoiMC4wLjAwMDAiLCJQIjoiV2luMzIiLCJBTiI6Ik1haWwiLCJXVCI6Mn0%3D%7C3000%7C%7C%7C&sdata=7eSRuVtR%2F9h42OW%2F8n2lNNu6BPD2qvOZYzA403rr1dI%3D&reserved=0)?> The trial protocol and CT registry contain little to no information regarding the present sub-study, and it is difficult to determine how much of the study was pre-specified based on the information currently available.

- **Yes. We have now included the Statistical Analysis Plan in the Supporting Information.**

3. Please confirm whether the primary results of the trial have been published.

- **The primary results of the CCG trial have not been published. They are under review.**

  4. As your trial had to undergo important modifications in response to extenuating circumstances, please complete the CONSERVE-CONSORT checklist and provide in your Supporting Information.

  When completing the checklist, please use section and paragraph numbers, rather than page numbers.
- **We have completed the CONSERVE-CONSORT checklist and added this to the Supporting Information (along with the initial CONSORT checklist, updated to use section and paragraph numbers instead of page numbers) and removed the STROBE checklist.**

  **Comments from the reviewers:**

  **Reviewer #1:** Thank you to the authors for addressing my previous comments well. I have no further issues to raise.
- **Thank you for your previous comments, which have strengthened the manuscript.**

  **Reviewer #2**: The revised paper has the same issues as the earlier submission.
  1)      Asymptomatic women with a Nugent score >=7 do not have a disease.  There is no study showing any benefit to treating women (or adolescents) in this scenario.  Regardless of what the study team thought, this is not the standard of care.  I can understand that if the IRB were presented with the information that it was a disease, they would want it treated.  If instead the IRB understood that there is no benefit to treatment of asymptomatic women with a vaginal gram stain score >=7, I suspect they would have felt differently.
- **We have added statements in the Methods (page 8, para 1) and Discussion (page 22, para 1) to further highlight that this differed from standard practice and therefore may not generalize to those who do not receive BV screening and treatment.**

2)      Because the research team's approach to handling asymptomatic women with a Nugent score >=7 this is clearly a deviation from the standard of care, I think this needs to be clearly stated in the paper.

- **We have added statements to the Methods and Discussion clearly stating this.**

3)      It is true that both arms were tested and treated equally for BV, but the response that this does not introduce bias misses the point.  It is a problem with generalizability.  The study did not test menstrual cup versus no menstrual cup.  Instead, it tested regular screening and treatment for BV (regardless of symptoms) versus regular screening and treatment of BV plus a menstrual cup.  It is not possible to generalize that the effect on BV would be the same in the absence of regular screening and treatment of BV in both arms.  I think that for the paper to be publishable, it would need to
a.      Clearly explain the two arms as: (1) cup plus screening and treatment of BV versus (2) screening and treatment of BV

- **We have added this to the Methods (page 8, para 1), and about generalizability in the Discussion.**

b.      In the discussion, clearly note that it may or may not be possible to generalize these findings to a population that is not receiving regular screening and treatment for both asymptomatic and symptomatic BV.  I.e. without doing the study, you cannot assume that menstrual cups alone would have the same effect.  It might be greater or less than the effect observed in this trial.

- **Thank you, as above, we have added this to the Discussion (page 22, para 1).**

4)      I'm puzzled by the authors response about randomization, given that the title of the study presents it as a nested cohort study within a cluster-randomized trial.  The title appears to present it as a cohort.  I actually agree that the randomization remains valid, so it may be the title that is the problem.  Of course it would not make sense to re-randomize.  However, it is a very small cRCT - I would still like to see the findings adjusted for the baseline difference in BV.  I'm not sure what the authors' response about this means - that BV at baseline is included in the baseline stream.

**🡪 Thank you, we have been in discussion with the Journal Editor to consider this. To prevent confusion, we have now changed the title and wording throughout to a nested “analysis” within cRCT.**

- **We have clarified on page 14, para 1 that baseline BV status and baseline STI status are included in both crude and multivariable adjusted analyses.**

  Reviewer #3: Mehta and colleagues' revised manuscript and response to reviewers sufficiently addressed my comments from my prior review, with one minor exception (first comment below). I also include a few comments related to minor typos I saw in the text that are important for the science and can be fixed quickly.
- **Thank you for the supportive comments and thoughtful review.**

Minor comments
Thank you for your response to my prior comment about baseline BV being different between the arms. Even though you adjust for baseline BV, please revise the text (line 289 in clean, 296 in track changes) to not say that baseline BV was similar/reflect the difference between arms.

- **We have updated the text as follows: Baseline STI and CST were similar between groups, and BV was somewhat increased among control arm participants (page 16, para 1).**

Line 211 in clean, 213 in track changes - "Quality and primer trimmed" should read "Quality filtered and primer trimmed" or something along those lines.

- **Thank you. We have made this correction.**

Make sure to reference Table 4 in Adjusted, Sub-group, and Sensitivity Analyses subsection of results.

- **Thank you – we have now referenced Table 4.**

The crude BV OR in table 3 is 0.76, but in the text it's 0.74. Please correct as needed.

- **Many thanks for this careful catch! We cross-checked with the output and the table 4 value is correct and we have now corrected the text.**

  Signed: Kayla A. Carter
