# Supplementary material for: Analysis of bacterial vaginosis, the vaginal microbiome, and sexually transmitted infections following the provision of menstrual cups in Kenyan schools: Results of a nested study within a cluster randomized controlled trial
Source: PLoS Med. 2023 Jul 25;20(7):e1004258. doi: 10.1371/journal.pmed.1004258 (PMC10368270; doi:10.1371/journal.pmed.1004258)
Supplement: Attachment — Submitted filename: Rebuttal Response R3 31May23.docx [file pmed.1004258.s008.docx]

Requests from Editors:

**We are grateful for these final edits to the manuscript and have made where possible, as follows:**

We ask you to amend the title to the following: "Analysis of bacterial vaginosis, the vaginal microbiome and sexually transmitted infections following provision of menstrual cups in Kenyan schools: Findings of a nested study within a cluster-randomized controlled trial".

- **We’ve made this change. However, we maintain the word “Results” rather than “Findings”, especially since we are changing the title to “Analysis” and results flow from analysis.**

Please move the trial registration number (line 17) to the end of the abstract.

- **This has been done.**

Please remove the information on study funding from the title page (in the event of publication this will appear in the article metadata, via entries in the submission form).

- **We have done this.**

At line 45, please make that "It is not known ..." or similar (reserving "We" for the authors). At line 48, that could become "We assessed ...".

- **This has been done.**

At line 58, please soften the language, and we suggest "In this study, we found that menstrual cups were ...".

- **Thank you for this. This sentence at line 58 is contextualization that notes findings from a prior systematic review and meta-analysis, not our current study (citations are provided in the text). We have clarified this now to state “Other studies have found that menstrual cups are a safe and cost-effective tool for menstrual hygiene management.”**

At line 59, we ask you to amend the text to "Further research should investigate the constitution of the vaginal microbiome and incidence of bacterial vaginosis in adolescent girls using menstrual cups".

- **We have added this sentence.**

At line 104, please refer to the CONSORT attachment by name here (see below).

- **We have done this.**

At line 428, please adapt the text to "... the study was not powered ...".

- **This has been done.**

At line 430, please soften the language to "We believe that our findings generalize to adolescent ...".

- **We have done this.**

At line 438, in a request from our academic editor, please amend the sentence beginning "These results generated ..." to "These results generated from a randomized study design suggest there are beneficial impacts of menstrual cups on the composition of the vaginal microbiome and a reduction in BV. "

- **We have done as required.**

Noting the description at line 445, the attached CONSORT checklist will need to be labelled "S1_CONSORT_Checklist" or similar and referred to as an individual supplementary file.

- **We have done this**

Please adapt the header for figure 1 to "Participant flow diagram" or similar.

- **We have done this.**

  Please convert journal names to non-italic text in the reference list.
- **This has been done.**

  Noting reference 1, please use the journal name abbreviation "PLoS ONE".
- **We have done this.**

Noting reference 29, please list up to 6 author names, followed by "et al.".

- **We have done this.**

Comments from Reviewers:

***** Reviewer #2:**

In my first two reviews, I restricted my comments to the most serious problems with the study design.  I did not try to make a comprehensive listing of things that would need to be changed for publication, as I wasn't recommending a resubmission.  In its current form, I still do not think the paper should be published.

The authors clearly want this to be the seminal paper that shows that menstrual cups reduce BV and increase Lactobacillus concentrations.  This is evident from their statements at the end of the abstract, in the author summary, and the conclusion.  For example, the conclusion states, "These results generated from a randomized study design provide strong evidence for beneficial effects of menstrual cups on the composition of the vaginal microbiome and reduction of BV."  I appreciate that the authors have added the statement, in the limitations, that the results may not be generalizable because screening and treatment for BV is not standard of care.  However, the fact that participants were screened and treated for BV means that the conclusions, as written, are not well supported by the data.  The paper is written as though it is a comparison of menstrual cups versus no menstrual cups, rather than a comparison of menstrual cups plus screening and treatment for BV vs. screening and treatment for BV.

- **We have softened the concluding statement.**

My summary of the conclusions, looking at the same data, is that in a study of screening and treatment for BV plus menstrual cups vs. screening and treatment of BV alone, the arm receiving screening and treatment of BV plus menstrual cups had lower BV and higher Lactobacillus crispatus.  As screening and treatment of BV in asymptomatic women with a vaginal Gram stain score >=7 is not recommended and would not be a realistic intervention for scale up in this setting, the findings point to the need for a trial comparing menstrual cups to no menstrual cups to determine whether the menstrual cup alone is a beneficial intervention.  The finding in the paper is interesting, but the authors are over-selling it as a definitive study showing the benefit of menstrual cups - this paper doesn't do that.  As it stands, the abstract, author summary, first paragraph of the discussion, and conclusion, in addition to most of the body text, obscure rather than illuminate what the study really examined, what it can teach us, and what it cannot.

- **We have taken this into account by reiterating that the conditions of BV and STI testing and treatment were the same across arms.**
- **Regarding provision of treatment to participants in this study:**
  - **Not all guidelines indicate treatment solely for those with symptoms.**
  - **Additionally, we considered guidelines in the context in which the study was taking place.**
  - **The British Association of Sexual Health and HIV guidelines, which we cite, advise the following under Management: “Women who do not volunteer symptoms may elect to take treatment if offered”.**
  - **In the IUSTI/WHO guidelines, which we also cite, the recommendation is also optional: “Optional: positive direct microscopy in women without symptoms.”**
  - **The decision to treat all participants with BV detected by Nugent score 7-10 was also based on the context of symptoms:**
    - **Symptoms have poor validity for Nugent score detected BV. Not only in our baseline findings (as cited), but also in our other studies of young adult women in Kenya (e.g., https://www.ncbi.nlm.nih.gov/pmc/articles/PMC7668344/), and those of others showing a substantial proportion of women with BV do not recognize symptoms as compared to clinical examination findings (e.g., review by https://www.ncbi.nlm.nih.gov/pmc/articles/PMC8015387/).**
    - **Even if symptoms were accurately recognized and reported by participants, for those who developed symptoms interim to study visits, there is limited opportunity outside of the study to confidentially seek diagnosis and treatment and to pay for diagnosis and treatment, creating clinical and ethical concerns for these minors.**
- **Therefore, given that symptoms are poorly recalled, and even if reported, are poorly predictive of BV, that there is little other opportunity for testing and treatment in this setting, and that normative body guidelines are inclusive of antibiotic treatment of “asymptomatic” BV, we treated the cases of BV detected among study participants.**
- **Regarding the possibility of doing a randomized trial of menstrual cups with BV as an outcome and not providing treatment to all cases of BV detected: As summarized above, the investigators did not find this to be an ethical option.**
- **Taking into account the reviewers concern that the current writing is unclear/obscures the study objectives and findings, we have edited the abstract, author summary, first paragraph of discussion and conclusion to better clarify this for readers.**

Major Comments:
1)      On lines 207-208, and again on lines 433-434, the authors explain that girls with a Nugent score of 7-10 were treated due to lack of correlation of reported symptoms with BV.  The statement that symptoms don't correlate with Gram stain score is true, but irrelevant, since treatment in this situation is not recommended.  This should be removed.

- **This statement remains relevant because it was part of the decision process to treat all cases of BV, as explained above.**

2)      Throughout the paper, including abstract, author statement, and body text, the authors should make it clear what the actual intervention and control conditions were, rather than trying to obscure this and use a single statement in the limitations.  Understanding this study design issue is critical to understanding the results - and the need for a definitive trial of cup vs. no cup.

- **We had no intent to obscure this, and have edited the manuscript including the abstract, author summary, methods, discussion, and limitations further to make this very clear to readers.**
